# A systematic review of the effectiveness of patient education through patient portals

**DOI:** 10.1093/jamiaopen/ooac085

**Published:** 2023-01-18

**Authors:** Adam M Johnson, Andrew S Brimhall, Erica T Johnson, Jennifer Hodgson, Katharine Didericksen, Joseph Pye, G J Corey Harmon, Kerry B Sewell

**Affiliations:** Department of Human Development & Family Science, East Carolina University, Greenville, North Carolina, USA; Department of Human Development & Family Science, East Carolina University, Greenville, North Carolina, USA; Department of Human Development & Family Science, East Carolina University, Greenville, North Carolina, USA; Department of Human Development & Family Science, East Carolina University, Greenville, North Carolina, USA; Department of Human Development & Family Science, East Carolina University, Greenville, North Carolina, USA; Department of Family Medicine, ECU Health, Greenville, North Carolina, USA; Laupus Library, East Carolina University, Greenville, North Carolina, USA; Laupus Library, East Carolina University, Greenville, North Carolina, USA

**Keywords:** personal health records, patient education

## Abstract

**Objective:**

The objective of this study was to systematically review all literature studying the effect of patient education on patient engagement through patient portals.

**Introduction:**

Patient portals provide patients access to health records, lab results, medication refills, educational materials, secure messaging, appointment scheduling, and telehealth visits, allowing patients to take a more active role in their health care decisions and management. A debate remains around whether these additional aids actually improve patient engagement and increase their ability to manage their own health conditions. This systematic review looks specifically at the effect of educational materials included in patient portals.

**Materials and Methods:**

In accordance with PRISMA guidelines, the literature search was mapped across 5 databases (PubMed, CINAHL, Scopus, PsychINFO, Embase), and implemented on June 2, 2020.

**Results:**

Fifty-two studies were included in the review. Forty-six (88.5%) reported rates of patient utilization of educational resources in the patient portal. Thirty (57.9%) shared patients’ perceptions of the usefulness of the education materials. Twenty-one (40.4%) reported changes in health outcomes following educational interventions through the patient portal. This review found that efforts are indeed being made to raise awareness of educational resources in patient portals, that patients are increasingly utilizing these resources, that patients are finding them useful, and that they are improving health outcomes.

**Conclusion:**

It seems that patient portals are becoming a powerful tool for patient education and engagement, and show promise as a means of achieving the quadruple aim of healthcare. Moving forward, research should establish more uniform methods of measurement in order to strengthen the literature surrounding the effectiveness of patient education through patient portals.

## INTRODUCTION

Since the Institute of Medicine (IOM) established new aims for an improved healthcare system, efforts have been made to make healthcare safe, effective, patient-centered, timely, efficient, and equitable.^1^ Patient engagement has been shown to help achieve the quadruple aim of healthcare, which is to: (1) improve patient outcomes, (2) improve patient experience, (3) reduce costs, and (4) improve the providers’ experience in their work.^2^*Patient education* is an effective way to increase *patient engagement* in their own health.^3^ Healthcare providers and organizations believe that using education to increase patient engagement will improve patients’ knowledge, skill, and confidence in managing their own health and health care.^3^^,^^4^

Current research shows that patients are interested in and want education and information about their health and how to manage it.^5^^,^^6^ They are increasingly turning to online sources for information about their conditions, especially when they feel providers fail to answer their questions.^7–9^ While the internet can be helpful^10^^,^^11^ sources such as YouTube, Wikipedia, Google, and social media platforms are unfiltered information sources and sometimes contain information that is either less accurate or inaccurate.^7^^,^^12^^,^^13^ This has problematic implications particularly for patients with lower health literacy, with research indicating they have a harder time distinguishing between reputable and unreputable sources.^14^

To address patients’ needs for health information and mitigate issues associated with poor quality information in many internet sites, healthcare providers, organizations and electronic health record (EHR) vendors created apps or digital resources to provide reputable education and tools to help patients manage their health.^15^ Some of these apps are standalone resources, while some called patient portals or personal health records (PHRs), are directly tethered or connected to the EHRs of providers and organizations.^15^ These tethered patient portals provide patients access to their health records, secure messaging, appointment scheduling, lab results, medication refills, educational materials, and telehealth visits, increasing patients’ ability to take an active role in their health care decisions and management.^15^ Healthcare providers can leverage patient portals to meet the rising demand for health education and information by providing reputable resources to patients online in a controlled and tailored manner.^7^^,^^8^^,^^11^ Currently, educational resources included in patient portals often include reading materials, videos, and links to reputable websites that are approved or created by the organization producing the patient portal.^15^

Previous reviews have studied patient portals generally, reviewing all the literature produced in recent years regarding their effect on patient empowerment,^16^^,^^17^ safety and quality of care,^18^ and utilization.^19–22^ In a meta-analysis, Neves et al^18^ found that patient portal use was associated with improved health outcomes for patients, as well as increased safety and quality of care. Alternatively, Ammenwerth et al^16^^,^^17^ found that patient portals had little to no effect on patient empowerment and health outcomes. Despite attempts to expand patient portals, patient use and adoption of these resources has been relatively low.^19^ To date, the percentage of patients who are registered to use their patient portal is typically less than 50% of the total patient population, and the number of patients who use it routinely is even lower.^23^ There appears to be a discrepancy between the resources provided and patients’ use of these resources. Zhao et al reviewed all literature surrounding barriers and facilitators of utilization and found that demographic factors such as age, race, and socioeconomic status can limit patients’ ability to access or use patient portals.^19^^,^^24^ A common barrier to accessibility is providing resources at a literacy level that the average patient can understand.^19^ For example, Chaet et al^20^ in their review, raised concern over the lack of patient portals that have resources in Spanish for Spanish speaking patients. Grossman et al^21^ reviewed all interventions used to increase utilization, and Beal et al^22^ reviewed the different ways researchers have used to track utilization. Current methods for increasing and measuring utilization have been widely varied and almost unique to the organization or research conducting the study, so there have been calls to analyze and standardize which practices are the most effective on both fronts in order to unify the field in a more common practice.^21^^,^^22^

While all these contributions have been valuable, no systematic reviews have been done to date to summarize the research regarding the use of patient education through patient portals. Just as with other aspects of patient portals, providers need to know about the effectiveness of education resources provided through patient portals, what factors determine patient use, and how to increase their effectiveness in the future. The objective of this study was to systematically review all literature studying the effectiveness of patient education through patient portals on patient engagement and ability to manage their health conditions.

### Research question

1. What is the effectiveness of educational resources within patient portals?
  - Are patients aware of the educational resources within the patient portal?
  - Are patients utilizing the educational resources?
  - Do the patients find the content useful?
  - Does use of patient education information in patient portals increase patients’ understanding of their own health conditions and improve their self-management or health outcomes?

## MATERIALS AND METHODS

The terms PHR and patient portal have become nearly synonymous in the research,^15^ so while both terms were included in our review’s search terms, we refer to these resources as patient portals throughout this review. The systematic review protocol and data underlying this article are available online via the Open Science Framework (https://osf.io/uey5c/?view_only=b3a792a77ab64fc8a5e5a39cec0cdfbd).

### Search strategy and screening

One author (GJCH), who is an information specialist/librarian, iteratively designed the search strategy using recommended controlled vocabulary and keywords for patient education and patient portals (see Table 1), in collaboration with 2 other authors (AMJ and KBS). The search was initially mapped in PubMed using MeSH terms and syntax and subsequently mapped to controlled vocabulary and syntax for the other identified databases (CINAHL, Scopus, PsychINFO, Embase). PubMed and Embase are standard databases for systematic reviews. The other 3 cover additional journals not indexed by PubMed and Embase but also focus on specific subject areas that were relevant to the research question. No limitations were placed on the search (ie, date, peer review, geography, or language). The date of the original search was June 2, 2020 All studies produced by the initial search were loaded into Endnote X9 (Clarivate Analytics, USA) for manual deduplication. After deduplication, the studies were loaded into Covidence (www.covidence.org), which identified additional duplicates missed during initial deduplication.

**Table 1.** Reproducible search terms for PubMed

(“Medical Records”[mh] OR “Patient Access to Records”[mh] OR “health record”[tiab] OR “health records”[tiab] OR “Patient portal”[tiab] OR “Patient portals”[tiab] OR “Patient Web Portal”[tiab] OR “Patient Web Portals”[tiab] OR “Patient Internet Portal”[tiab] OR “Patient Internet Portals”[tiab] OR “Patient access to records”[tiab] OR ((electronic[tiab] OR automated[tiab] OR medical[tiab]) AND record*[tiab]) OR EHR[tiab] OR EMR[tiab] OR PHR[tiab] OR e-PHR[tiab]) AND (“Patient Participation”[mh] OR “health literacy”[mh] OR “consumer health information”[mh] OR “consumer health informatics”[mh] OR “patient education as topic”[mh] OR “Patient Involvement”[tiab] OR “Patient Empowerment”[tiab] OR “Patient Participation”[tiab] OR “Patient Activation”[tiab] OR “Patient Engagement”[tiab] OR “health literacy”[tiab] OR “patient education”[tiab] OR “patient guideline”[tiab] OR “patient guidelines”[tiab] OR “teaching material”[tiab] OR “teaching materials”[tiab] OR “instructional material”[tiab] OR “instructional materials”[tiab] OR “educational materials”[tiab] OR “educational material”[tiab] OR “consumer health information”[tiab] OR “consumer health informatics”[tiab] OR “consumer health materials”[tiab])

Two reviewers (AMJ and ETJ) independently screened all titles and abstracts against inclusion and exclusion criteria (see Table 2). Conflicts were resolved by discussion until consensus was reached. Articles that passed the initial screening were included in a second round of screening, where studies underwent duplicate full-text review by 2 authors (AMJ and ETJ) to determine their inclusion to the final review. Any discrepancies between the reviewers were resolved by discussion.

**Table 2.** Criteria for inclusion and exclusion

Inclusion	Exclusion
Tethered personal health records or patient portals only (PHR’s as an extension of the EHR system of a healthcare organization). Educational resources provided by the organization to the patients through the tethered patient portal system.	Not tethered to provider/organization's EHR (ie, online registry creates record of patient-reported outcomes, app not from provider) No education about health conditions Application for providers, not a patient portal Product meets criteria, but does not answer the questions of this review (either focused on an aspect other than educational resources, or did not include patient data) Based on provider communication (providers use patient portal to send education. Not included in the patient portal) Not a patient portal (apps can be tethered, draw information, without allowing patients access back to their record) Duplicate study missed during initial screen

### Data extraction and synthesis

The PRISMA guideline for reporting systematic reviews was followed while conducting this study (https://prisma-statement.org/PRISMAStatement/Checklist). Data extraction was done using Covidence and Microsoft Excel. Extracted data elements included: author(s) last name(s), year of publication, country and language of publication, patient portal used, population size and characteristics, sampling method, study design, outcomes, and findings. Data extraction was performed by the primary investigator (AMJ) and confirmed by another author (ETJ). Data were synthesized using narrative synthesis methods.^25^ One author (AMJ) synthesized findings and one author (ASB) checked the results and provided recommendations for any needed changes.

### Risk of bias and interrater reliability

Although risk of bias assessment is recommended for systematic reviews, study methods varied considerably among included studies. Since of risk of bias assessment instruments are created for specific study designs (see the variety of checklists here: https://casp-uk.net/casp-tools-checklists/), providing a unified risk of bias assessment using just 1 or even 2 risk of bias tools was not possible. Interrater reliability analysis using the Kappa statistic was performed to determine consistency among the screeners. Interrater reliability for the title and abstract screening was found to be Kappa = 0.275, and title abstract screening for the full text review was found to be Kappa = 0.499. These measurements are considered to be fair and moderate, respectively.^26^

## RESULTS

**Figure 1.**
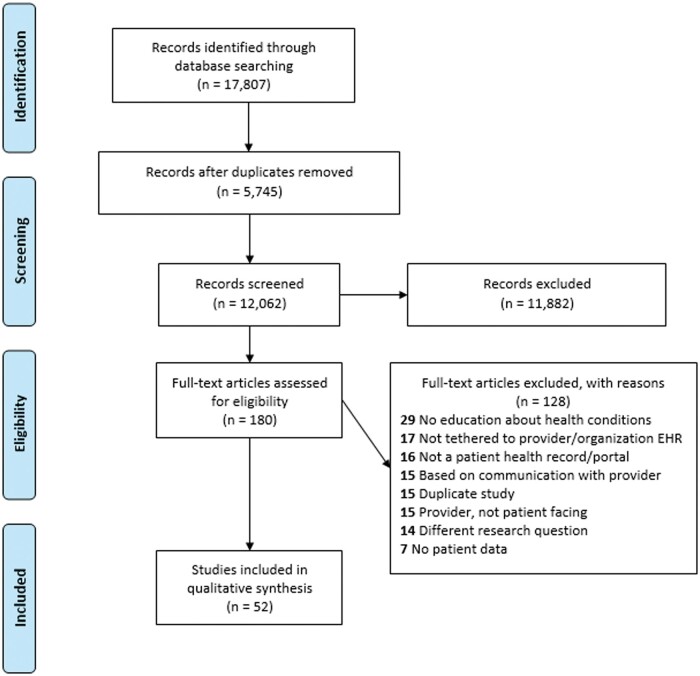
PRISMA flowchart of the inclusion/exclusion process.

The initial search retrieved 17,807 records across the 5 identified databases (PubMed—5347, CINAHL—3018, Scopus—2152, PsycInfo—3383, Embase—3018). Following manual (EndNote) and machine-driven deduplication (Covidence), 12,062 unique articles remained for title and abstract screening. Following title and abstract screening, 180 articles were retained for full text review. Fifty-two articles were retained for full inclusion in this review. The PRISMA flow diagram (Figure 1) provides a record of the article selection process for the review.

Due to the large number of studies included (*n* = 52), a master table with information from all studies is included as Supplementary Material. Only a minority of studies included demographic information on participants, so a synthesis of that data was not included in this review. Results are organized by the research questions, with smaller tables describing the articles relevant to each of the 4 subquestions guiding the review. Of the studies included, 36 (69.2%) were based on US healthcare systems and 16 (30.8%) were based on other countries. Forty-four of the patient portal systems (84.6%) were utilized in outpatient settings, while 9 (17.4%) were for inpatient treatment (one was utilized in both, and so double counted). Forty-three (82.7%) provided education for the patient, 8 provided education for both the patient and the caregiver (15.2%), and one targeted only the caregivers (2%). The populations targeted in the included studies varied across several health conditions (ie, diabetes, cancer, pregnancy, etc.). Another important finding was that 40 of the studies (77%) were published between 2013 and 2020, with only one published before 2000,^27^ reflecting that patient portals are still a relatively new resource being developed.

### Question 1—are patients aware of the educational resources within the patient portals?

**Figure 2.**
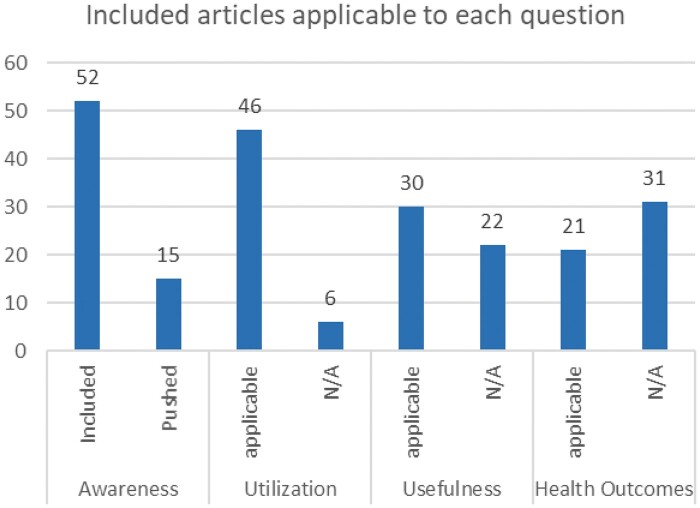
Number of included articles relevant to each of the 4 research questions. Results are sectioned according to research question. “Applicable” represents the number of articles included in the review, which relate to each of the 4 questions. N/A indicates the number of included articles which did not include results relevant to each question.

The primary factor determining whether an article was included was its use of patient education within the patient portal. As a result, all 52 studies had some educational features as a portion of their patient portal and each made attempts to inform their patients about its availability (see Figure 2). Of these studies, 15 (28.8%) had content pushing features, meaning that the patient portal automatically linked patients with relevant educational resources based on their medical information, such as diagnoses.^28–42^ While these studies confirm that patients were notified of the educational resources available, especially those who were enrolled in the 15 studies relying on push notifications, it does not necessarily provide insight into the awareness of the typical patient who is not enrolled in an ongoing study measuring the use of patient portals.

### Question 2—are patients utilizing the educational resources?

While all included patient portal studies had educational resources and made patients aware of them, utilization of these resources remained low. Of the 52 studies included in the review, 46 (88.5%) reported on patient’s utilization of the educational resources (see Figure 2). This data was obtained in a variety of ways, including: (1) survey data relying on patient self-report (*n* = 16; 30.8%); (2) required utilization for pilot studies (*n* = 11; 21.2%), (3) qualitative statements simply saying that resources were used (*n* = 10; 19.2%); and (4) metadata audits of patient portal systems and how often resources were clicked on or accessed (*n* = 9; 17.3%).

The most common form of data collection (survey data) revealed that, according to patients’ self-report, utilization rates varied from as low as 20%^43^ to 95%^42^ with an overall average of 47% across 12 studies (see Table 3).^27^^,^^30^^,^^32^^,^^36^^,^^41–48^

**Table 3.** Rates of utilization

Authors	Utilization
Arcia (2017)	75% (12/16)
Cameron et al (2016)	47% (55/116)
Cho et al (2019)	42% (84/201)
Groen et al (2016)	89% (33/37)
Jones et al (1992)	24% (17/70)
Kelly et al (2017)	61% (111/181)
Naveethan et al (2017)	47% (99/209)
Roelofsen et al (2014)	27% (110/405)
Steiner et al (2017)	20% (43/216)
Warrington et al (2019)	78% (276/354)
Woollen et al (2016)	85% (12/14)
Zhang et al (2016)	95% (19/20)

*Note*: Parentheses show the number of patients who reported utilizing the patient portal education over the total sample size. The total number of patients who utilized patient portal education in these studies was 871/1839 (47%).

This finding excludes the 11 studies whose utilization rates were 100% because participants were required to use it as part of the study.^39^^,^^49–56^ The variability in utilization rates seems to be associated with sample size, as studies with smaller samples typically experienced higher utilization rates. There also appears to be some discrepancy between desire for resources and the actual rate at which participants use them. Patients reported wanting educational resources included in the patient portal between 84%^5^^,^^6^ and 89%^57^ of the time, and planned on using them “very often.”^58^ It is also worth noting that 78% of caregivers also indicated that they wanted educational resources.^5^ Although many participants reported wanting educational resources, and felt they would use them frequently, the data indicates that expressed desire for patient portal-based educational resources does not equate with an equally high level of use of those resources.

Metadata audits of the patient portal software reported the number of times educational resources were accessed over varying amounts of time^23^^,^^31^^,^^59–65^ but the nondescript nature of the audit made it hard to interpret how frequently educational resources were utilized by individual patients. However, one of the studies specifically reported that patients who accessed educational resources through the patient portal made up only 42% of registered patient portal users, and 10% of the entire patient population of that site.^23^

### Question 3—do the patients find the content useful?

Only 30 of the 52 studies included in the review included some aspect that measured patients’ perception of the usefulness of educational content (see Figure 2). Based on the review of those articles, it appears that patients find educational resources in patient portals to be extremely useful. Like utilization rates, these reports were received using a variety of methods, including: (1) surveying the study sample to see what percentage of participants perceived education resources as useful (*n* = 13, 43%), (2) qualitative statements simply stating that patients thought content was useful (*n* = 9, 30%), (3) having participants report level of usefulness using Likert scale measurements (*n* = 6, 20%), and (4) utilizing validated measures of usefulness (*n* = 2, 7%).

Of the 4 different styles of measurement, the 2 most informative were the survey studies reporting perceived usefulness by the percentage of participants and those which relied on Likert scales to quantify the degree of usefulness, both of which showed that patients thought the educational resources within the patient portal were very useful (see Table 4).^5^^,^^27^^,^^29^^,^^30^^,^^39^^,^^42^^,^^45^^,^^46^^,^^48–51^^,^^53^^,^^54^^,^^57^^,^^58^^,^^66–68^ Two studies used the Net Promoter Score^44^ and the System Usability Scale^65^ to measure patient’s perceptions of usefulness, and both reported that patients found educational resources included in the patient to be very useful. While this only makes up 7% of the included studies measuring perceptions of usefulness, they are noteworthy because they represent a shift in the field to more standardized methods of measurement. The remaining 9 simply stated that patients thought the resources were useful, often with quotations from qualitative studies.^40^^,^^43^^,^^55^^,^^59^^,^^61^^,^^63^^,^^69–71^

**Table 4.** Patient’s report of education resources’ usefulness

Authors	Percentage	Authors	Likert
Benhamou (2011)	74% (13/18)	Martinez et al (2018)	5.8/7
Warrington et al (2015)	42% (5/12)	Baek et al (2018)	4.23/5
Groen et al (2016)	69% (26/37)	Ahmed et al (2020)	4.3/5
Day et al (2019)	78% (7/9)	Arcia (2017)	4.7/5
Yeh et al (2008)	70% (35/50)	Hefner et al (2017)	4.5/5
Fricton et al (2008)	70% (128/182)	Fiks et al (2014)	8.4/9
Hess et al (2006)	71% (15/21)		
Wiljer et al (2006)	79% (37/46)		
Wiljer et al (2010)	98% (123/125)		
Woollen et al (2016)	86% (12/14)		
Jones et al (1992)	84% (59/70)		
Kelly et al (2017)	68% (61/90)		
Zhang et al (2016)	96% (19/20)		

*Note*: Parentheses show the number of patients reporting patient portal education as useful over the total sample size. The total number of patients who said patient portal education was useful across these studies was 540/689 (78%).

Likert scale shows the average score of patient reports of usefulness of the patient portal education resources. A score above the midpoint of the scale (ie, <3/5, <4/7, <5/9) indicates patients reporting that the education was useful.

### Question 4—does use of patient education information in patient portals increase patients’ understanding of their own health conditions and improve their self-management or health outcomes?

The use of patient education resources provided through the patient portal had a positive effect on patient outcomes. Of the 52 articles included in the review, 21 (40.4%) reported on the association between patient portals and patient self-management and health outcomes (see Figure 2). While each of these included studies used patient portals that featured patient education resources among other tools, they did not differentiate the individual contribution of education on patient outcomes, reporting results instead as the effects of the whole intervention. Patient outcomes were measured in 3 ways, including: (1) improvement in self-management of health conditions (*n* = 12, 57%), (2) changes in patient lab values (*n* = 5, 24%), and (3) adoption of desired health behaviors (*n* = 4, 19%).

The most common measure of patient outcomes, self-management of health, was determined in 2 different ways; quantitative measures that scored improvement (*n* = 6) and qualitative reports from patients (*n* = 6). The 6 studies that used quantitative outcomes found that use of educational resources in the patient portal were associated with significant increases in: (1) patient activation^45^; (2) knowledge of conditions^33^^,^^56^; (3) self-management^42^; (4) adoption of desired health behaviors^33^; (5) decision making^55^; and (6) decreased anxiety.^68^ The patients in the qualitative self-report studies indicated they felt that education through the patient portal: (1) increased their knowledge of their health^30^^,^^46^^,^^48^^,^^61^^,^^63^; (2) increased their confidence in their ability to take care of themselves or those in their care^30^^,^^40^^,^^46^^,^^61^^,^^63^; (3) improved safety during and after hospitalization^46^^,^^48^; (4) improved decision making and ability to communicate with healthcare providers^46^; and (5) decreased anxiety around treatment and prognosis.^40^^,^^48^ It should be noted that 3 studies also found improvements that, while positive, were nonsignificant in areas of self-efficacy,^68^^,^^72^ adherence,^72^ knowledge,^4^ and health behaviors such as physical exercise.^45^

As for improvements in health behaviors and lab values, 4 studies reported significant improvements in patients adopting positive health behaviors such as receipt of vaccination,^32^ use of effective contraception,^37^ medication compliance,^33^ and decision to undergo a preventative screening procedure.^50^ Changes in patient lab values (ie, HgA1c) following use of educational resources were positive, though not always significantly so. Two studies associated significant improvements^66^^,^^70^ in intervention groups who were given educational resources, while 3 found improvements that were not significantly better than control groups.^4^^,^^36^^,^^44^ In each of these cases, nonsignificant results still showed slight positive changes and were often presented alongside significant findings in other aspects of patient health, so it seems that use of educational resources in the patient portal is generally associated with improvements in patient outcomes.

## DISCUSSION

This review sought to evaluate the effectiveness of educational resources within patient portal systems by determining availability and patients’ awareness of resources, their utilization and perceived usefulness of those resources, and the effect of those resources on patient outcomes. In response to the first 2 research questions of this study, many providers and healthcare organizations have developed or are utilizing patient portals to provide more accurate and reliable sources of information in response to patients’ increased interest in their own healthcare, and are making efforts to increase patient awareness of these resources.^5^^,^^6^ The main discussion point, however, is how to increase utilization of educational resources through patient portals in the general population. In response to the second two research questions of this study, educational resources provided through patient portals useful (see Table 4), and when used, they had a significant impact on patient outcomes.^32^^,^^33^^,^^37^^,^^50^^,^^66^^,^^70^ The methods used to measure usefulness and patient outcomes varied widely, and creating more uniform methods of measurement will be the focus of discussion.

### Questions 1 and 2

While this review indicates that patient education through patient portals improves patient experiences and patient outcomes, utilization of educational resources is low (see Table 3)^23^ even after patients are made aware of them. These findings highlight a common finding in effectiveness research. Utilization rates of educational resources are potentially inflated because attempts to study patient portals require that participants are enrolled in studies where they are being asked, as part of the study, to use the resources. This was especially true in pilot studies where utilization rates were 100% because participants were required to use them. However, in studies which simply measured rates of use (ie, metadata studies) utilization rates of both patient portals and educational resources are relatively low. These findings present an interesting dilemma for professionals in this field that needs to be addressed. It may be that patients from the general population are still not aware of these features and therefore they remain underutilized. Strategies for increasing use by the average patient need to be developed. A couple of strategies that seem to make a difference, based on this review, is including additional resources, like patient navigators, or automating connection of patients with relevant resources. Considering that this technology is still relatively new, there is hope that patient utilization will increase as patients become more aware of its availability. Like many new technologies, utilization rates might continue to be low for the average American until a tipping point occurs that shifts use from early adopters (those who are either technologically savvy or heavily invested in their health) to part of the mainstream culture. Efforts to raise this awareness will be essential if patient portals are ever going to realize the full potential these educational resources have to increase patient health. Also, it is important to recognize that, given the rapid pace of technological advances, new technologies will continue to expand and develop, thus, requiring research to evolve with it.

### Questions 3 and 4

Current research suggests that patients found educational resources provided through patient portals useful (see Table 4), and when used, they had a significant impact on patient outcomes.^32^^,^^33^^,^^37^^,^^50^^,^^66^^,^^70^ While this review focuses on patient perceptions, it is also worth noting that Fricton et al^5^ found that 70% of caregivers thought it was useful too. Given that caregiver engagement can have enormous impacts on patient outcomes, this finding is notable.^73^ The variability of methods of measurement for utilization, usefulness, and impact of patient education included in this review may be due to the fact that research regarding patient education through patient portal systems is still in its infancy. Most of the articles included in the review (77%) were published between 2013 and 2020, because patient portals themselves are a relatively new technology. Up to this point, most research has focused on development, design, implementation, and beta testing new systems and resources, and so have used a wide variety of methods and measures of effectiveness. While these studies have added to the existing literature the fact that they are measuring different variables makes it hard to establish a consistent message regarding utilization rates and effectiveness. One benefit of a systematic review is it provides a comprehensive summary of the literature that exists around a specific topic and highlights some of the potential gaps. One finding this review highlights is that patients who are using the educational resources found in patient portals are finding them helpful. How helpful is hard to determine because researchers have typically used a variety of different methods to measure both utilization and effectiveness. Different measurements make it difficult to develop a cohesive argument regarding the effectiveness of these resources. Future research in this area should include a Delphi study to establish the best method of standardized measurement to help strengthen the argument surrounding patient portals and their effectiveness, as survey data does not seem to reliably measure use in the general poplulation^23^ and measures such as the Net Promoter Score^44^ do not specifically measure utilization or outcomes, but rather satisfaction. Until a specific study is done to provide a more definitive recommendation for standardize measurement of patient portal utilization, it seems that system audits of actual use, as used in the study done by Ancker et al^23^ provide the most accurate measure of utilization, and that Likert scales seem to be the most uniform way to measure perceived usefulness.

### Limitations

This systematic review screened and included a large quantity of literature regarding broad topics of patient engagement, patient education, and health records, which may have contributed to the moderate interrater reliability and may limit the reproducibility of this study. While it had several strengths there is an important limitation to consider. The majority of articles that match our criteria are from the United States (69.2%). There are several possible explanations for this outcome. First, all of the authors on this project are from the United States and therefore may be more familiar searching systems based on literature published predominantly in the United States. Second, the search was limited to patient portals that were tethered. Other countries may have studies related to patient education that operate differently enough to not meet the inclusion criteria for this review (ie, Australia’s patient-owned record, which is not tethered to an organization). Lastly, it is also possible that most of the articles were from the United States because the regulatory and reimbursement-based mechanisms noted above create an environment in which some healthcare systems provide patient portals that do not support the addition of features beyond access to the patient’s medical record.

### Implications for future research

Now that patient portals are more common, future researchers studying the effect of education through patient portals should seek to use more uniform methods of measurement so that results can be easily compared between studies and systems. We are already witnessing the beginning of this trend, with some studies using validated measures of effectiveness rather than just participant self-report.^44^^,^^65^ What’s more, after beta testing educational resources in patient portals with small sample populations, studies should move to observe rates of utilization in the general population so that we can observe their impact in everyday contexts, rather than in controlled settings.^23^

In response to the issue of low utilization, future research should study the effect of interventions (ie, pushing education or patient navigators) on patient portal patient education utilization. Providers and organizations can raise awareness and utilization by programing patient portals to push, or connect, relevant education to patients. Relying on push notifications may help providers avoid the existing problem that is happening where a library of educational resources lies dormant while patients are seeking unreliable sources to answer many of their health care questions because they do not know where else to look.^74^^,^^75^ Personalized or patient-relevant education can be pushed to patients based on diagnosis,^28^^,^^29^^,^^35^^,^^38^ lifespan stage,^30^^,^^76^ prevention for at-risk populations,^32^^,^^37^ or any other personal health information.^31^^,^^42^ This can be done by providers, sometimes known as patient navigators,^36^ but can also be automated to reduce costs.^39–41^ It may be beneficial for future research to focus more on specific strategies that help increase utilization rates.

A word of caution. One of the problems often discussed about patient portals are barriers to adoption.^24^^,^^77^ Disparities have been found in patient use of patient portals and educational resources by age, race, and socioeconomic status.^24^ While patient portals present a low risk of harm to patients if misused, researchers should consider the risk of widening health disparities by providing yet another tool to privileged populations,^78^ and thus widening what has been dubbed “the digital divide”^24^ between privileged and underprivileged populations. Simple interventions such as providing visual summaries of personal health information, such as charts or picture descriptions,^79^ have been found to help neutralize disparities in health literacy.^77^ Future researchers need to be cognizant of issues of privilege and oppression that may be accentuated by interventions with patient education through patient portals and other rising technology in healthcare.

## CONCLUSION

This review found that efforts are being made to raise awareness of educational resources in patient portals, that patients are increasingly utilizing these resources, that patients are finding them useful, and that they are improving health outcomes. It seems that patient portals are becoming a powerful tool for patient engagement and show promise as a means of achieving the quadruple aim of healthcare.^2^ Continued efforts need to be made to raise awareness of patient educational resources in patient portals so that patients utilize these resources to increase their knowledge, skills, and confidence for managing their own health and health care.^3^

## AUTHOR CONTRIBUTIONS

All authors contributed to the study conception and design. Search terms and databases were selected by AMJ, GJCH, and KBS, searches were run and results uploaded to Covidence by GJCH. The screening of abstracts and full text review was carried out by AMJ and ETJ. AMJ extracted and organized the data, which was then confirmed by ETJ. AMJ and ASB drafted the manuscript. JH, KD, and JP served as committee members on AMJ’s dissertation committee, and reviewed and helped edit the manuscript as part of that role.

## SUPPLEMENTARY MATERIAL

Supplementary material is available at *JAMIA Open* online.

## CONFLICT OF INTEREST STATEMENT

None declared.

## Supplementary Material

ooac085_Supplementary_Data

## Data Availability

The data underlying this article are available in the Dryad Digital Repository.
