# Supplementary material for: A systematic review of the effectiveness of patient education through patient portals
Source: JAMIA Open. 2023 Jan 18;6(1):ooac085. doi: 10.1093/jamiaopen/ooac085 (PMC9847535; doi:10.1093/jamiaopen/ooac085)
Supplement: ooac085_Supplementary_Data [file ooac085_supplementary_data.docx]

| Appendix 1. *Reproducible search terms for each database* | | |  |
| --- | --- | --- | --- |
| Pubmed | | ("Medical Records"[mh] OR "Patient Access to Records"[mh] OR "health record"[tiab] OR "health records"[tiab] OR "Patient portal"[tiab] OR "Patient portals"[tiab] OR "Patient Web Portal"[tiab] OR "Patient Web Portals"[tiab] OR "Patient Internet Portal"[tiab] OR "Patient Internet Portals"[tiab] OR "Patient access to records"[tiab] OR ((electronic[tiab] OR automated[tiab] OR medical[tiab]) AND record*[tiab]) OR EHR[tiab] OR EMR[tiab] OR PHR[tiab] OR e-PHR[tiab]) AND ("Patient Participation"[mh] OR "health literacy"[mh] OR "consumer health information"[mh] OR "consumer health informatics"[mh] OR "patient education as topic"[mh] OR "Patient Involvement"[tiab] OR "Patient Empowerment"[tiab] OR "Patient Participation"[tiab] OR "Patient Activation"[tiab] OR "Patient Engagement"[tiab] OR "health literacy"[tiab] OR "patient education"[tiab] OR "patient guideline"[tiab] OR "patient guidelines"[tiab] OR "teaching material"[tiab] OR "teaching materials"[tiab] OR "instructional material"[tiab] OR "instructional materials"[tiab] OR "educational materials"[tiab] OR "educational material"[tiab] OR "consumer health information"[tiab] OR "consumer health informatics"[tiab] OR "consumer health materials"[tiab]) | |
| Psychinfo | ( ( DE “medical records” OR DE “electronic health records” OR DE “client records” ) OR TI ( (“health record” OR “health records” OR “Patient portal” OR “Patient portals” OR “Patient Web Portal” OR “Patient Web Portals” OR “Patient Internet Portal” OR “Patient Internet Portals” OR “Patient access to records” OR ((electronic OR automated OR medical) AND record*) OR EHR OR EMR OR PHR OR e-PHR) ) OR AB ( (“health record” OR “health records” OR “Patient portal” OR “Patient portals” OR “Patient Web Portal” OR “Patient Web Portals” OR “Patient Internet Portal” OR “Patient Internet Portals” OR “Patient access to records” OR ((electronic OR automated OR medical) AND record*) OR EHR OR EMR OR PHR OR e-PHR) ) ) AND ( ( DE “Client Participation” OR DE “Health literacy” OR DE “Client education” ) OR TI ( (“Patient Involvement” OR “Patient Empowerment” OR “Patient Participation” OR “Patient Activation” OR “Patient Engagement” OR “health literacy” OR “patient education” OR “patient guideline” OR “patient guidelines” OR “teaching material” OR “teaching materials” OR “instructional material” OR “instructional materials” OR “educational materials” OR “educational material” OR “consumer health information” OR “consumer health informatics” OR “consumer health materials”) ) OR AB ( (“Patient Involvement” OR “Patient Empowerment” OR “Patient Participation” OR “Patient Activation” OR “Patient Engagement” OR “health literacy” OR “patient education” OR “patient guideline” OR “patient guidelines” OR “teaching material” OR “teaching materials” OR “instructional material” OR “instructional materials” OR “educational materials” OR “educational material” OR “consumer health information” OR “consumer health informatics” OR “consumer health materials”) ) ) | |  |
| CINAHL | ( (MH “Medical Records” OR MH “Patient Access to Records”) OR TI ( (“health record” OR “health records” OR “Patient portal” OR “Patient portals” OR “Patient Web Portal” OR “Patient Web Portals” OR “Patient Internet Portal” OR “Patient Internet Portals” OR “Patient access to records” OR ((electronic OR automated OR medical) AND record*) OR EHR OR EMR OR PHR OR e-PHR) ) OR AB ( (“health record” OR “health records” OR “Patient portal” OR “Patient portals” OR “Patient Web Portal” OR “Patient Web Portals” OR “Patient Internet Portal” OR “Patient Internet Portals” OR “Patient access to records” OR ((electronic OR automated OR medical) AND record*) OR EHR OR EMR OR PHR OR e-PHR) ) ) ) AND ( ( MH “consumer participation” OR MH “Health literacy” OR MH "Patient Education+" OR MH “Consumer health information”) OR TI ( (“Patient Involvement” OR “Patient Empowerment” OR “Patient Participation” OR “Patient Activation” OR “Patient Engagement” OR “health literacy” OR “patient education” OR “patient guideline” OR “patient guidelines” OR “teaching material” OR “teaching materials” OR “instructional material” OR “instructional materials” OR “educational materials” OR “educational material” OR “consumer health information” OR “consumer health informatics” OR “consumer health materials”) ) OR AB ( (“Patient Involvement” OR “Patient Empowerment” OR “Patient Participation” OR “Patient Activation” OR “Patient Engagement” OR “health literacy” OR “patient education” OR “patient guideline” OR “patient guidelines” OR “teaching material” OR “teaching materials” OR “instructional material” OR “instructional materials” OR “educational materials” OR “educational material” OR “consumer health information” OR “consumer health informatics” OR “consumer health materials”) ) ) ) | |  |
| SCOPUS | TITLE-ABS ( ( ( "health record"  OR  "health records"  OR  "Patient portal"  OR  "Patient portals"  OR  "Patient Web Portal"  OR  "Patient Web Portals"  OR  "Patient Internet Portal"  OR  "Patient Internet Portals"  OR  "Patient access to records"  OR  ( ( electronic  OR  automated  OR  medical )  AND  record* )  OR  ehr  OR  emr  OR  phr  OR  e-phr ) )  AND  ( ( "Patient Involvement"  OR  "Patient Empowerment"  OR  "Patient Participation"  OR  "Patient Activation"  OR  "Patient Engagement"  OR  "health literacy"  OR  "patient education"  OR  "patient guideline"  OR  "patient guidelines"  OR  "teaching material"  OR  "teaching materials"  OR  "instructional material"  OR  "instructional materials"  OR  "educational materials"  OR  "educational material"  OR  "consumer health information"  OR  "consumer health informatics"  OR  "consumer health materials" ) ) ) | |  |
| EMBASE | ('medical record'/exp OR 'health record':ti,ab OR 'health records':ti,ab OR 'patient portal':ti,ab OR 'patient portals':ti,ab OR 'patient web portal':ti,ab OR 'patient web portals':ti,ab OR 'patient internet portal':ti,ab OR 'patient internet portals':ti,ab OR 'patient access to records':ti,ab OR ((electronic:ti,ab OR automated:ti,ab OR medical:ti,ab) AND record*:ti,ab) OR ehr:ti,ab OR emr:ti,ab OR phr:ti,ab OR 'e phr':ti,ab) AND (('patient participation'/exp OR 'health literacy'/exp OR 'patient education'/exp OR 'consumer health information'/exp OR 'consumer health informatics'/exp) AND 'patient involvement':ti,ab OR 'patient empowerment':ti,ab OR 'patient participation':ti,ab OR 'patient activation':ti,ab OR 'patient engagement':ti,ab OR 'health literacy':ti,ab OR 'patient education':ti,ab OR 'patient guideline':ti,ab OR 'patient guidelines':ti,ab OR 'health plan implementation':ti,ab OR 'health plan implementations':ti,ab OR 'teaching material':ti,ab OR 'teaching materials':ti,ab OR 'instructional material':ti,ab OR 'instructional materials':ti,ab OR 'educational materials':ti,ab OR 'educational material':ti,ab OR 'consumer health information':ti,ab OR 'consumer health materials':ti,ab) | |  |
